# Acid-Hydrolysis-Assisted Cellulose Nanocrystal Isolation from *Acacia mearnsii* de Wild. Wood Kraft Pulp

**DOI:** 10.3390/polym16233371

**Published:** 2024-11-29

**Authors:** Daniel Tavares de Farias, Jalel Labidi, Cristiane Pedrazzi, Darci Alberto Gatto, Pedro Henrique Gonzalez de Cademartori, Carline Andréa Welter, Gabriela Teixeira da Silva, Tielle Moraes de Almeida

**Affiliations:** 1 Laboratório de Química da Madeira (LAQUIM), Forest Science Department, Universidade Federal de Santa Maria—UFSM, Av. Roraima 1000, Santa Maria 97105-900, RS, Brazil; 2 Chemical and Environmental Engineering Department, University of the Basque Country UPV/EHU, Plaza Europa, 1, Donostia-San Sebastián 20018, Guipuzcoa, Spain; 3 Postgraduate Program in Materials Science and Engineering, Federal University of Pelotas, Pelotas 96010-610, RS, Brazil; darcigatto@yahoo.com; 4 Department of Forestry Engineering and Technology (UFPR), Universidade Federal do Paraná—UFPR, Av. Prefeito Lothário Meissner, 632—Jardim Botânico, Curitiba 80210-170, PR, Brazil; pedrocademartori@gmail.com; 5 Programa de Pós-Graduação em Ciências Farmacêuticas, University of Santa Maria—UFSM, Av. Roraima 1000, Santa Maria 97105-900, RS, Brazil; tiellefarm@gmail.com

**Keywords:** ultrasonic treatment, yield, *Acacia mearnsii*, cellulose nanocrystals

## Abstract

Cellulose nanocrystals (CNC) receive great attention for their physical and optical properties, high surface area, high tensile strength, rigidity (Young’s modulus up to 140 GPa), and ease of surface modification. However, controlling the properties of CNC is still challenging, given the wide variety of pulp sources and the complexity of finding suitable processing conditions. In the present study, acid hydrolysis efficiently isolated CNC from wood *Acacia mearnsii* brown kraft pulp (AMKP). Initially, the AMKP was delignified by the treatment with acidified sodium chlorite. The Acacia mearnsii kraft pulp obtained was then subjected to acid hydrolysis with sulfuric acid at concentrations of 50 to 58% 45 °C for 60 min. The hydrolysate was sonicated in an ultrasonic processor for 30 min. The chemical composition was determined by Fourier transform infrared spectroscopy (FTIR), crystallinity by X-ray diffraction (XRD), zeta potential by Zetasizer ZS equipment, thermal stability by thermogravimetric analysis (TGA), and morphology by transmission electron microscopy (TEM) to verify the effect of acid concentration on the yield and properties of CNC. The optimization of the isolation process demonstrated that the maximum yield of 41.95% can be obtained when AMWP was hydrolyzed with sulfuric acid at a concentration of 54%. It was possible to isolate CNC with a crystallinity index between 71.66% and 81.76%, with the onset of thermal degradation at 240 °C; zeta potential of −47.87 to 57.23 mV; and rod-like morphology, with lengths and widths between 181.70 nm and 260.24 nm and 10.36 nm and 11.06 nm, respectively. Sulfuric acid concentration significantly affected the yield of acid hydrolysis, allowing the isolation of CNC with variable dimensions, high thermal stability, high crystallinity index, and great colloidal stability in aqueous medium.

## 1. Introduction

In recent decades, increasing attention has been directed to ecological and environmentally friendly materials due to the environmental pressure caused by synthetic polymers that can remain in the environment for hundreds of years. This has resulted in a priority global demand to accelerate the transition to using raw materials from sustainable sources. In this context, lignocellulosic biomass has been highlighted as a source of biodegradable, renewable polymers available in nature and at low cost.

Cellulose nanocrystals (CNC), extracted from cellulose fibers that make up lignocellulosic biomass, have gained deserved prominence as a natural biopolymer for combining good optical properties, low thermal expansion, and easy chemical modification surface, being biocompatible and renewable [1] as well as suitable for the synthesis of green products in a variety of fields, such as the packaging, paper, biomedical, food emulsions, cosmetics, and cementitious material industries [2]. The global market value of plant nanocellulose, including CNC and cellulose nanofibrils (CNF), is expected to reach USD 2.712 billion by 2025, with an annual growth rate of 18.80% per year between 2018 and 2025 [3].

The typical method of CNC preparation is the hydrolysis of cellulose fibers by strong mineral acid, most commonly H_2_SO_4_, HCL [3], H_3_PO_4_, HBr [4], and HNO_3_ [5]. Binary mixtures such as HCl/HNO_3_ [6], H_2_SO_4_/H_2_C_2_O_4_ [7], H_2_SO_4_/CH_3_COOH [8], and H_2_SO_4_/HCOOH (Wang et al., 2021) are also reported to be efficient in CNC isolation. Sulfuric acid has been extensively studied in hydrolysis methods to obtain nanocrystal suspensions with high electrosphere stability due to the negative charges of sulfate ester groups that bind OH as CNC are released from the selective hydrolysis of cellulose microfibrils amorphous regions [9].

The performance of CNC as reinforcement material in nanocomposite films with improved barrier properties [10]; pickering emulsion stabilizer [11]; reinforcing agent in hydrogels for tissue engineering [12]; and composites and foams for automotive, aerospace, and civil construction [13], among other advanced applications, is directly related to the structural properties, surface chemistry, morphology, and crystallinity of CNC. These are inherent to cellulose sources and the isolation method of nanocrystals.

For this reason, the efficient isolation of CNC with specific morphological, structural, and surface chemical characteristics has been challenging, given the abundant diversity of lignocellulosic biomass and the need to formulate the optimal processing parameters. A source of lignocellulosic biomass that has not yet been investigated for obtaining CNC is the genus Acacia, which occupies the fourth position in the ranking of trees planted in Brazil, with about 160 thousand hectares of commercial plantations that are mainly concentrated in the southern region of the country [14], being an important source of wood for energy, cellulosic pulp, and paper and tannin.

Given this scenario, the present work investigated the effect of sulfuric acid concentration during the hydrolysis of delignified kraft pulp from *Acacia mearnsii* on the chemical, structural, morphological, and thermal properties of isolated CNC.

## 2. Materials and Methods

### 2.1. Materials

*Acacia mearnsii* brown kraft wood pulp (AMKP) with a kappa number of 11.1 was produced at the Wood Chemistry Laboratory of the Universidade Federal de Santa Maria (LAQUIM/UFSM) following the procedure described by Giesbrecht, B. et al. (2022) [15]. In short, the commercial wood chips obtained from the SPA were subjected to kraft pulping. The cooking operations were carried out in a REGMED AUE/20 rotary digester with four 1 L reactor cells, electrically heated and equipped with a thermometer and pressure gauge, allowing four cooking operations in a single batch, using 200 g of OD wood chips (oven-dried). To prepare the white liquor, 98% sodium hydroxide (NaOH) and 50% sodium sulphide tetrahydrate (Na_2_S) were used. The extraction method for cellulose nanocrystals was selected based on a comprehensive review of the relevant literature. The chemical reagents used in the experiment were sodium chlorite (80% Sigma Aldrich, St. Louis, MI, USA), sodium acetate ag (Synth), glacial acetic acid ag (Synth), and sulfuric acid (95.0–98.0% ag Vetec).

### 2.2. Delignification of Brown Kraft Pulp Acacia mearnsii Wood (AMKP)

AMKP delignification was performed the procedure described by Wise [16]. An absolutely dry 25 g sample of AMKP was transferred to an Erlenmeyer flask containing 3.5 g sodium chlorite, 3.5 g sodium acetate, 25 drops of glacial acetic acid, and 400 mL of distilled water. The mixture was kept in a water bath at 80 °C for 1 h and manually stirred at intervals of 10 min. After the reaction time had elapsed, the material was washed with excess running water to remove unreacted chemicals and air-dried for 12 h. This process was repeated twice until achieving high whiteness of the material, indicating the removal of non-cellulosic components. The delignified pulp was identified as AMWP.

### 2.3. Isolation of AMWP CNC

CNCs were isolated from AMWP using a modified version of the classical sulfuric acid hydrolysis method [9]. Hydrolysis was conducted with sulfuric acid at concentrations of 50%, 52%, 54%, 56%, and 58%, using 200 mL of the acid for each 10 g of AMWP, under vigorous and constant mechanical stirring for 1 h in a water bath at 45 °C. Once the reaction time was completed, the hydrolysis was stopped by adding a volume of ice-cold distilled water equivalent to eight times the volume of acid used in the reaction.

After cooling, the solution was centrifuged at 10 °C and 15,000 rpm. The supernatant was removed, and the sediment was diluted with distilled water. This procedure was repeated four times to obtain a cloudy suspension containing CNC. The CNC suspension was dialyzed (cellulose membrane; 10,000 Da) against distilled water for six days, until the pH of the aqueous medium reached neutrality, to remove free acid, and then was sonicated in an ultrasonic probe processor at 30% amplitude for 30 min in an ice bath.

The CNC suspensions resulting from sulfuric acid hydrolysis at the concentrations of 50%, 52%, 54%, 56%, and 58% were named CNC-50, CNC-52, CNC-54, CNC-56, and CNC-58, respectively. The yield of the sulfuric acid hydrolysis process was determined by gravimetry, collecting an aliquot of each CNC suspension, which was subsequently dried in an oven at 60 °C for 24 h. The percentage yield was calculated by the percentage ratio between dry CNC and the initial mass of AMWP. The analysis was performed in three replicates, the results were subjected to analysis of variance, and the means were subsequently compared by Fisher’s LSD test (*p* < 0.05).

### 2.4. Fourier Transform Infrared Spectroscopy (FTIR)

Fourier transform infrared spectroscopy (FTIR) allows for the detecting of the frequency of chemical bonds and identifying the chemical composition of cellulose pulps (AMKP, AMWP) and cellulose nanocrystals extracted under different conditions. The Shimadzu Prestige FTIR equipment was used by the direct transmittance method using the KBR chip technique, with 45 scans in the reading range of 400 to 4500 cm^−1^.

### 2.5. ZETA Potential and DLS

The zeta potential and DLS of the CNC were determined by electrophoretic mobility in Zetasizer ZS equipment (Malvern Instruments, Malvern, UK). The CNC suspensions at 0.005% concentration in ultrapure water were sonicated in a probe ultrasonic processor at 750 W power, 30% amplitude, for 5 min in an ice bath and kept at rest for 48 h before being analyzed. Each analysis was performed with three replicates, the results were subjected to analysis of variance, and the means were subsequently compared by Fisher’s LSD test (*p* < 0.05).

### 2.6. X-Ray Diffraction (XRD)

The AMKP, AMWP pulps, and CNC crystal structure were investigated in an X-ray diffractometer model Miniflex 300 (Rigaku, Tokyo, Japan). Diffractograms were obtained at room temperature under Cu Ka radiation (λ = 1.54051 Å), a power source with 30 kV and 10 mA, and Step mode, with a scan speed of 0.5 s and scan step of 0.03° in angular range from 5 to 100° (2θ). The crystallinity indices (Ic) of all samples were calculated by deconvolution of the peaks observed in the diffractograms, taking a Gaussian distribution function as the format of the amorphous (2θ~12°18°) and crystalline (2θ~20°24°) peaks. The areas under the crystalline and amorphous peaks were estimated after baseline correction using the OriginLab 8 software, and the Ic was calculated by Equation (1), as described by Xing et al. (2018).

(1) $$Ic(\%)=\left(\frac{Crystalline\;zone}{Crystalline\;zone+Amorphous\;zone}\right)\times 100$$

### 2.7. Thermogravimetric Analysis (TGA)

The thermal stability of the AMKP and AMWP pulps and the CNC was investigated using the TGA-1000 equipment brand Navas Instrument. A 50 mg lyophilized sample was heated from room temperature to 1000 °C, with a heating rate of 10 °C/min, in a nitrogen gas environment at the flow rate of 1 L/min.

### 2.8. Morphological Analysis

The morphology of CNC was determined by transmission electron microscopy (TEM). Aqueous dispersions of 0.005% CNC were dripped onto carbon-coated transmission electron microscope grids, contrasted with 2% uranyl acetate dried for 24 h, and analyzed using the Joel-JEM 1200EX-II (JEOL Ltd., Tokyo, Kyoto, Japan) transmission electron microscope operating at a voltage of 80 kV. Representative images obtained in bright field mode were selected to measure the length and width of 100 CNC of each sample by digital image analysis of the Image-J software. The results were submitted to analysis of variance, and the means were subsequently compared by Fisher’s LSD test (*p* < 0.05).

## 3. Results and Discussion

### 3.1. Chemical Composition of AMKP and AMWP Pulps and CNC

**Figure 1:**
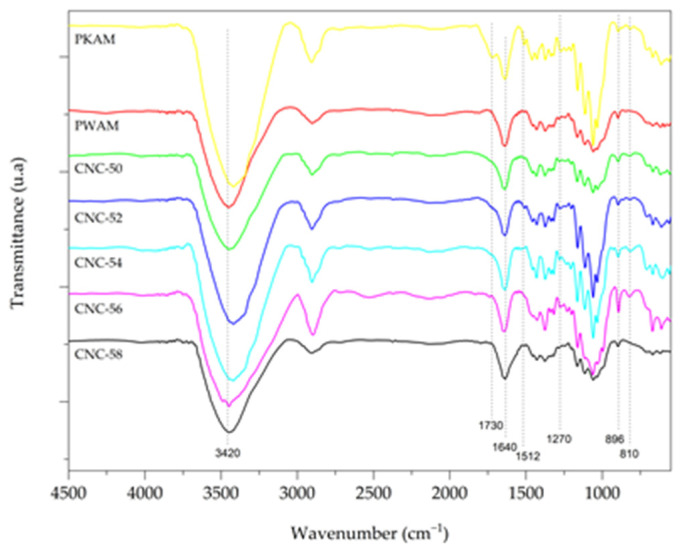
*Acacia mearnsii* brown kraft pulp (AMKP); *Acacia mearnsii* delignified kraft pulp (AMWP); and cellulose nanocrystals isolated by 50% (CNC-50), 52% (CNC-52), 54% (CNC-54), 56% (CNC-56), and 58% (CNC-58) sulfuric acid hydrolysis.

The FTIR spectra of the AMKP and AMWP pulps and CNC (Figure 1) were used to investigate their functional groups to examine the changes in their chemical composition that occurred after the delignification and isolation of the CNC.

The FTIR spectra of all pulps and CNC showed characteristic peaks of absorption of pure cellulose, as expected. The peaks at 896 cm^−1^ and 3420 cm^−1^ present in all spectra can be attributed to the typical cellulose structure and occur, respectively, due to the β-glycosidic bonds of the glucose ring and the elongation of the cellulose hydroxyl groups. The peaks at 1640 cm^−1^ are associated with adsorbed water [17,18].

Functional groups characteristic of hemicelluloses and lignin were verified in the AMKP curve due to its lignocellulosic structure. The bands in 1730 cm^−1^ and 1512 cm^−1^ are characteristic of the elongation of the C=O bond into carbonyl, ester, and acetyl groups in the xylose component of hemicellulose and lignin, and aromatic groups of lignin, respectively [19]. The disappearance of these peaks in the AMWP curve is the primary difference between the FTIR AMKP and AMWP curves, indicating that the delignification process efficiently removes the non-cellulosic components of AMKP.

The vibrations of OH and CH groups at 3420 cm^−1^ and 2900 cm^−1^ were observed in all FTIR curves of the CNC, indicating the presence of functional groups typical of wood cellulose [20]. The 1640 and 896 cm^−1^ bands are typical features of the C–O and C–H elongation of glycosidic bonds in cellulose [20] and were observed in all CNC FTIR curves.

The primary difference between the spectra of the CNC can be observed at the peak 810 cm^−1^ present only in the CNC-56 spectrum, which occurs as a result of esterification of the cellulose surface hydroxyl groups, with sulfate ions during the sulfuric acid hydrolysis [21]. The absence of this peak in the CNC-50, CNC-52, CNC-54, and CNC-58 spectra may have occurred due to the low sulfate content unable to be detected by FTIR.

### 3.2. CNC Isolation Process Yield

**Figure 2:**
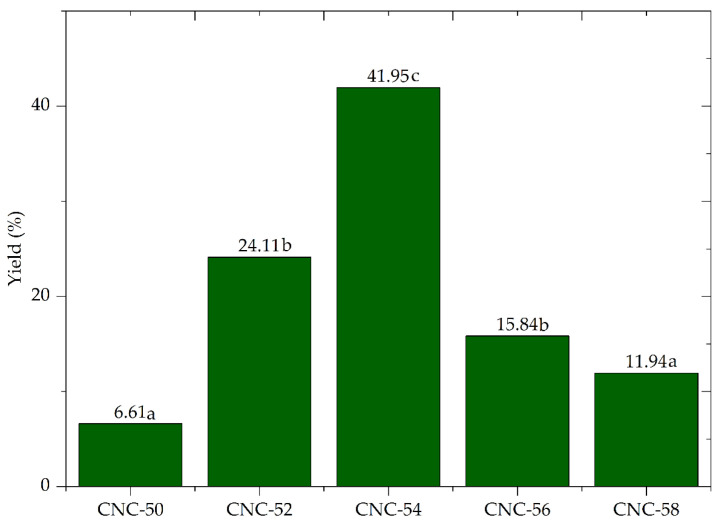
Average yield of the cellulose nanocrystals isolation process from the *Acacia mearnsii* delignified kraft pulp by sulfuric acid hydrolysis at 50% (CNC-50), 52% (CNC-52), 54% (CNC-54), 56% (CNC-56), and 58% (CNC-58). Values followed by equal letters did not differ statistically (*p* < 0.05) by Fisher’s test.

Figure 2 shows the yield values of CNC isolation with different concentrations of sulfuric acid. The increase in hydrolysis acid concentration from 50 to 54% resulted in an increase in process yield from 6.61% to 41.95%, with a subsequent decrease in yield to 11.94%, with an increase in hydrolysis acid concentration to 58%.

The H_2_SO_4_ in a 50 to 52% contraction range could not hydrolyze cellulose. Average yield values of 6.61% and 24.11% were verified for CNC-50 and CNC-52, respectively. The yield of 41.91% was achieved when the acid concentration was increased to 54% (CNC-54). However, the increase in acid concentration above 54% intensified the depolymerization of rapidly hydrolyzed cellulose chains, resulting in a small number of nanocrystals in CNC-56 (15.84%) and CNC-58 (11.94%).

These changes in the yield of CNC according to variation of acid concentration suggest that H_2_SO_4_ at 54% is the best condition to obtain high CNC yield of *A. mearnsii* at the moderate temperature of 45 °C. The acid concentration has a strong effect on the yield of CNC, and minor adjustments in the concentration of sulfuric acid can maximize the yield and reduce process cost [22,23,24].

### 3.3. Zeta Potential and Dynamic Light Scattering (DLS)

Zeta potential is often measured to determine the colloidal stability of suspended particles in dilute systems. Absolute values of zeta potential above 30 mV indicate that the electrostatic interactions in that system can maintain the suspensions stable for long periods.

However, due to low electrostatic repulsion between particles, particles tend to flocculate and aggregate when zeta potential measurements are inferior to 30 mV. Table 1 shows the mean zeta potential values of the isolated CNC from PWAM with different acid concentrations.

The mean absolute zeta potential values for CNC-50, CNC-52, CNC-54, CNC-56, and CNC-58 were 20.47, 44.63, 47.87, 57.23, and 49.30 mV, respectively. The concentration of H_2_SO_4_ used to hydrolyze the cellulose fibers of the pulp of *A. mearnsii* significantly affected the zeta potential values of the CNC isolates. The mean zeta potential values gradually increased as the concentration of H_2_SO_4_ used to hydrolyze cellulose fibers increased. These results show that it is possible to modulate the zeta potential values to obtain cellulose nanocrystals with desirable sulfate ion surface charge values for specific applications.

Dynamic light scattering (DLS) analysis is used to determine the equivalent hydrodynamic diameter of CNC from the diffusion coefficient. This is calculated assuming that the particles analyzed are theoretically spherical and move by means of Brownian motion. And even if the CNCs are shaped like needles, the DLS provides a size estimate based on the diffusion behavior of the particles in a solution [25]. The hydrolysis of sulfuric acid at different concentrations produced nanoparticles with equivalent mean diameter values (Table 1).

The sulfuric acid concentrations studied significantly affected the DLS values. The mean diameter of the CNCs ranged from 98.56 nm (CNC-54) to 417.60 nm (CNC-50). Nanocrystals CNC-52, CNC-56, and CNC-58 presented mean diameters of 139.73 nm, 137.10 nm, and 145.53 nm, respectively.

The acid hydrolysis applied to PWAM in the experimental conditions studied did not occur homogeneously. This can be explained by the different acid concentrations used and the number of fines generated by the successive delignification treatments applied to the raw material, causing different levels of accessibility of the acid inside the cellulose fiber.

### 3.4. Crystallinity

**Figure 3:**
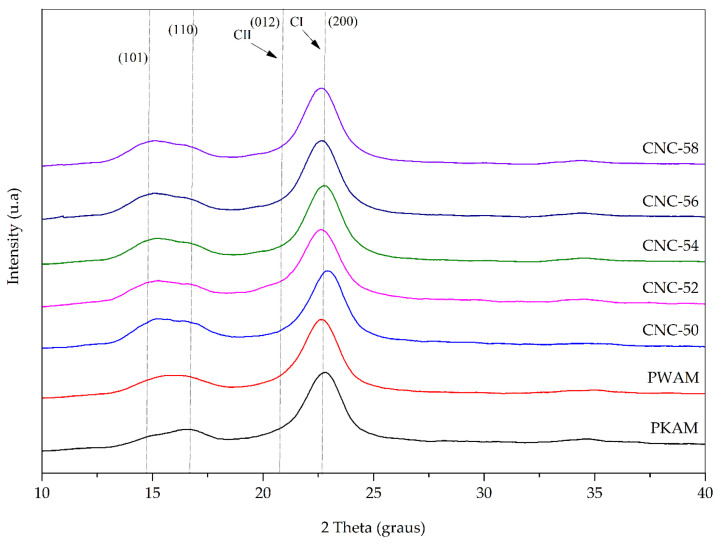
XRD standard of *Acacia mearnsii* brown kraft pulp (AMKP); *Acacia mearnsii* delignified kraft pulp (AMWP); and cellulose nanocrystals isolated by 50% (CNC-50), 52% (CNC-52), 54% (CNC-54), 56% (CNC-56), and 58% (CNC-58) sulfuric acid hydrolysis.

The crystallinity of AMKP, AMWP, and CNC was investigated by the X-ray diffraction technique (Figure 3). The diffractograms revealed typical peaks of type I allomorphic cellulose at 2θ = 14.5°, 16.8°, and 22.6°, and they refer to the crystalline planes (101), (110), and (200), respectively, which predominantly compose the cellulosic structure of plants with secondary growth.

After acid hydrolysis of PKWM, the same X-ray diffraction peaks were recorded for CNC-50, CNC-52, CNC-54, CNC-56, and CNC-58 diffractograms. This indicates the predominant presence of Iß cellulose. These crystallographic planes were also observed in the delignified pulp of *A. mangium* and its nanocrystals isolated by hydrolysis with sulfuric acid [26], as occurs in CNC isolated from cotton by sulfuric, hydrochloric, and phosphoric acid hydrolysis [27]. The crystallinity indices (Ic) calculated from the diffractograms in Table 2 indicate that the successive chemical treatments applied to the cellulose substrate increased crystallinity.

The delignification treatment with acidified sodium chlorite raised the pulp Ic from 33.22% (AMKP) to 37.42% (AMWP). This may be associated with removing non-cellulosic compounds such as hemicellulose and lignin. These results corroborate the findings discussed in the previous session, in which the characteristic bands of hemicellulose and lignin present in AMKP disappeared in the FTIR spectrum of AMWP, confirming the efficiency of delignification in obtaining a high-purity cellulosic pulp, consequently increasing crystallinity due to the removal of these amorphous components.

There was a 29.02% increase in crystallinity compared to PWAM when acid treatment was employed using H_2_SO_4_ at 50%, obtaining a CNC with 48.28% Ic (CNC-50). It was possible to verify an increase in the Ic of 62.93%, 71.66%, 76.80%, and 81.76% for the varying concentrations of H_2_SO_4_ for 52% (CNC-52), 54% (CNC-54), 56% (CNC-56), and 58% (CNC-58), respectively. The increase in Ic in 69.34% of CNC-58 compared to CNC-50 was due to the greater aggressiveness of the treatment, which was able to break the intramolecular and intermolecular bonds of the amorphous regions of the cellulose, releasing the individualized cellulose nanocrystals.

### 3.5. Thermal Stability

**Figure 4:**
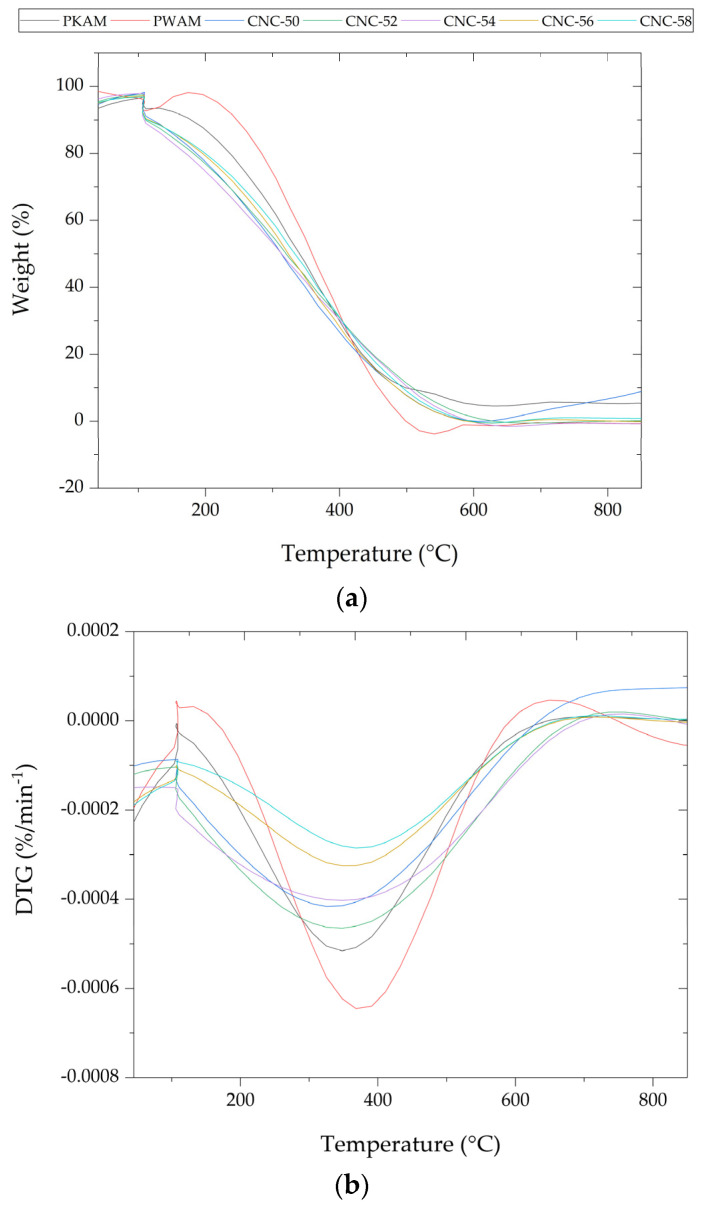
(**a**) Weight (%) and (**b**) differential thermogravimetric curves (DTG) of *Acacia mearnsii* brown kraft pulp (AMKP); *Acacia mearnsii* delignified kraft pulp (AMWP); and cellulose nanocrystals isolated by 50% (CNC-50), 52% (CNC-52), 54% (CNC-54), 56% (CNC-56), and 58% (CNC-58) sulfuric acid hydrolysis.

The thermal stability of AMKP, AMWP, and CNC as a function of temperature was investigated by thermogravimetric analysis (TGA) and differential thermogravimetric curves (DTG), as presented in Figure 4.

An initial mass loss of about 7% was observed in the AMKP and AMWP mass loss curves at 109 °C. It corresponds to moisture evaporation and can be visualized more accurately in the DTG curves. A new sharp peak was observed at 305 °C in the mass loss curve of AMKP due to hemicelluloses. This low thermal stability and early degradation is due to the acetyl groups that make up hemicellulose [28].

The degradation temperature increased to 368 °C after the delignification treatment, as observed in the PWAM DTG. This sharp increase in degradation temperature occurred due to the removal of hemicellulose and lignin by delignification. The presence of the peak was due to cellulose pyrolysis [29]. The thermogravimetric profiles obtained from all CNC samples presented two degradation stages that can be observed in the mass loss and DTG curves of Figure 4.

The first stage of degradation was verified between 220 °C and 240 °C. The first significant mass loss occurred for CNC samples in this temperature range. A notable decrease in cellulose degradation temperature can be observed compared to the AMWP DTG curve. This thermal degradation behavior around 230 °C was also verified for CNC isolated by the sulfuric acid hydrolysis of eucalyptus delignified kraft pulp [9].

The second stage of CNC degradation was observed between 430 °C and 450 °C. No significant variation was observed when comparing the thermogravimetric curves, suggesting that acid hydrolysis conducted at 45 °C, at a reaction time of 60 min, and H_2_SO_4_ (50–58%) had no noticeable effects on the thermal stability of CNC isolated from *A. mearnsii*.

In addition, the degradation temperature of the CNC of the present study was higher in relation to the CNC isolated from the bleached cellulosic pulp of the species *E. benthamii*, *E. globulus*, *E. smithii*, and one *E. nitens* × *E. globulus* hybrid, which occurred from 300 °C to 400 °C [30], and CNC extracted from blends of eucalyptus bleached kraft pulp (Eucalyptus globulus and Eucalyptus nitens, which occurred in the temperature range of 230–385 °C [9].

### 3.6. Transmission Electron Microscopy (TEM)

Transmission electron microscopy results show that cellulose nanocrystals morphology is similar to rods or needles, typical of cellulose nanocrystals extracted by sulfuric acid hydrolysis. The morphology of CNC is similar to the morphology of nanocrystals obtained by acid hydrolysis of mixed acids H_2_SO_4_/oxalic acid [7], H_2_SO_4_/formic acid [8], and HCL [6]. Average values of 398.83 nm, 280.5 nm, 260.24 nm, 236.48 nm, and 181.70 nm, respectively, and widths of 27.82 nm, 20.54 nm, 11.06 nm, 10.76 nm, and 10.36 nm were observed for the average length of the cellulose nanocrystals CNC50, CNC52, CNC54, CNC56, and CNC58.

The length and width values of the CNC were statistically compared (*p* < 0.05) with each other to evaluate the effect of acid concentration on the dimensions of the nanocrystals. Thus, a significant difference was observed between the nanocrystal samples, except when comparing the length values of CNC52 with CNC54. Most combinations of width values showed a difference at the 5% significance level, and only the comparison between CNC56 with CNC58, CNC54 with CNC56, and CNC52 with CNC58 showed no significant difference.

**Figure 5:**
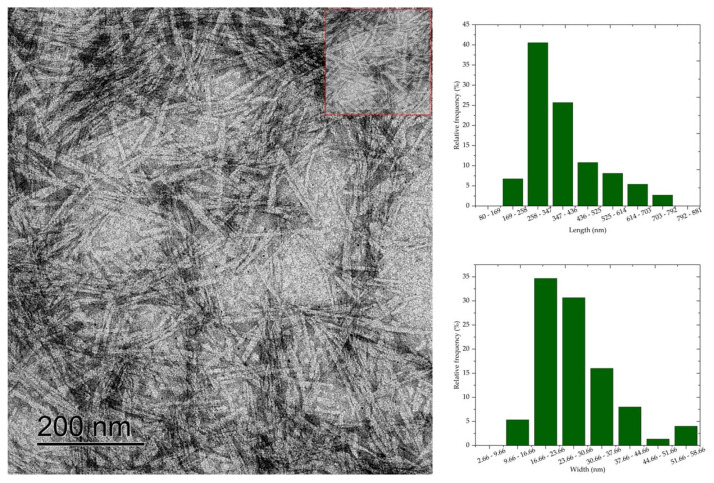
Cellulose nanocrystals isolated from *Acacia mearnsii* delignified kraft pulp by hydrolysis with 50% sulfuric acid at 45 °C and 60 min. The CNC are highlighted in red in the figure.

**Figure 6:**
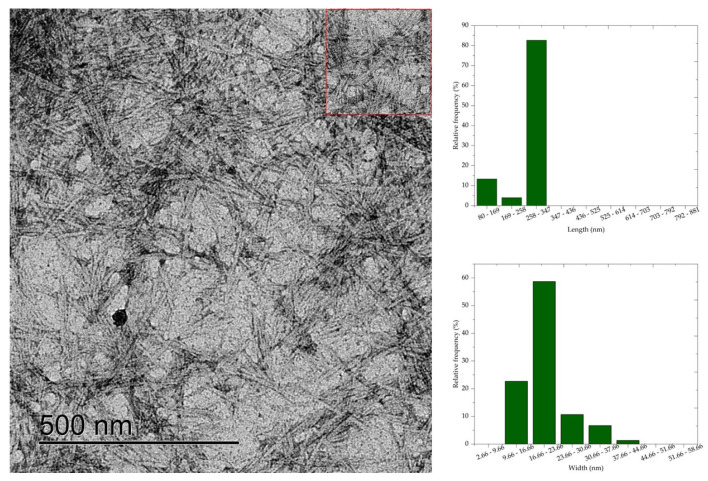
Cellulose nanocrystals isolated from *Acacia mearnsii* delignified by hydrolysis with 52% sulfuric acid at 45 °C and 60 min. The CNC are highlighted in red in the figure.

**Figure 7:**
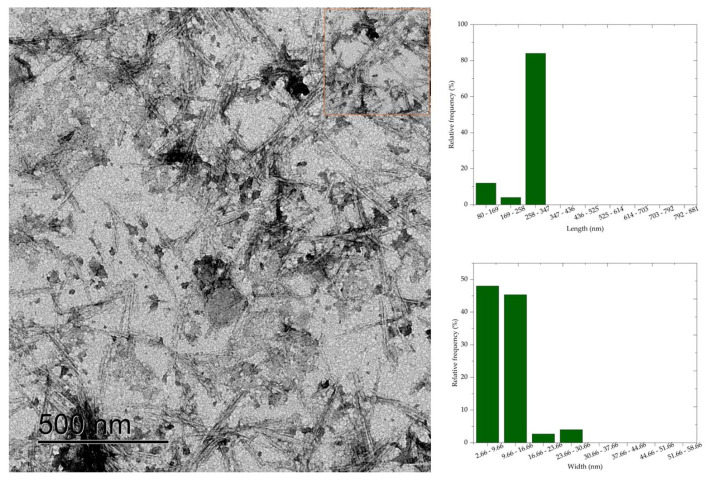
Cellulose nanocrystals isolated from *Acacia mearnsii* delignified by hydrolysis with 54% sulfuric acid at 45 °C and 60 min. The CNC are highlighted in red in the figure.

**Figure 8:**
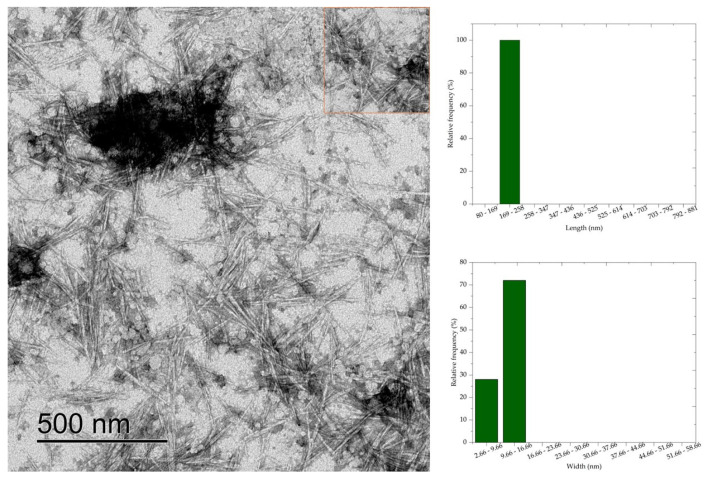
Cellulose nanocrystals isolated from *Acacia mearnsii* delignified by hydrolysis with 56% sulfuric acid at 45 °C and 60 min. The CNC are highlighted in red in the figure.

**Figure 9:**
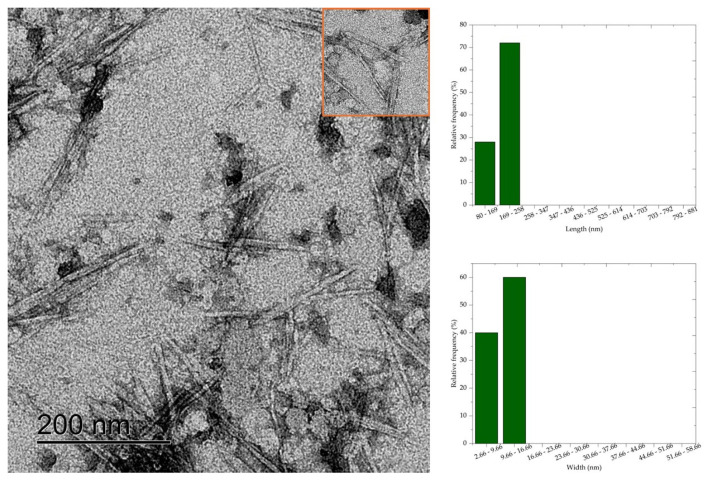
Cellulose nanocrystals isolated from *Acacia mearnsii* delignified by hydrolysis with 58% sulfuric acid at 45 °C and 60 min. The CNC are highlighted in red in the figure.

Comparing the micrographs of Figure 5, Figure 6, Figure 7, Figure 8 and Figure 9, it is observed that the length measurements of CNC50 and CNC52 were distributed in values greater than 258 nm, indicating the presence in the long and wide nanocrystals due to the width measurements above 9.66 nm. The length of the nanocrystals CNC54, CNC56, and CNC58 were concentrated in classes 258–347 nm, 169–258 nm, and 80–169 169–258, respectively, with widths between 2.66 and 16.66 nm.

Sulfuric acid hydrolysis at concentrations above 54% isolated cellulose nanocrystals with smaller length and width compared to nanocrystals isolated with acid at 50 and 52% concentrations. These differences in dimensions indicate that the depolymerization of cellulose was insufficient with sulfuric acid hydrolysis reactions at 50% and 52% concentrations, resulting in long nanocrystals, less homogeneous than the nanocrystals obtained at higher concentrations. The authors of [6] observed that nanocellulose particles isolated from bleached eucalyptus wood kraft pulp were similar to fibrils at low sulfuric acid concentrations (<56%), and the increase in acid concentration generated short CNC due to cellulose depolymerization influenced by the severity of acid hydrolysis.

## 4. Conclusions

In recent years, a multitude of CNC extraction techniques have been explored for various biomasses. This research investigated the characteristics of CNC extracted by sulfuric acid hydrolysis. The concentration of sulfuric acid significantly affects the yield of acid hydrolysis, where sulfuric acid concentrations above 56% result in excessive cellulose degradation. It was possible to isolate CNC with variable dimensions, high thermal stability, high crystallinity index, and significant colloidal stability in an aqueous medium by adjusting the sulfuric acid concentration between 54 and 58% at 45 °C and a reaction time of 60 min.

The maximum yield of 41.95% can be achieved by adjusting the sulfuric acid concentration to 54% at 45 °C and reaction time of 60 min. Under these conditions, the CNC has a crystallinity index of 71.66%, a zeta potential of 47.87 mV, and a hydrodynamic diameter of 98.56 nm.

## 5. Challenges and Future Perspectives

*Acacia mearnsii* biomass has a high commercial value and has gained prominence in the forestry sector due to its quality and productivity. However, there is still little research into the derivatives of its cellulose pulp. Therefore, future studies should explore new techniques for extracting nanocellulose from this biomass; optimize and evaluate new processes; and assess the technical, economic, and environmental feasibility of integrating *Acacia mearnsii* biomass into new biorefinery areas.

## Figures and Tables

**Table 1:** Mean values of zeta potential and nanoparticle size distribution by dynamic light scattering of cellulose nanocrystals isolated by sulfuric acid hydrolysis at 50% (CNC-50), 52% (CNC-52), 54% (CNC-54), 56% (CNC-56), and 58% (CNC-58).

Sample	Zeta Potential (mV)	DLS (nm)
CNC-50	−20.47 a	417.60 A
CNC-52	−44.63 b	139.73 B
CNC-54	−47.87 c	98.56 C
CNC-56	−57.23 d	137.10 D
CNC-58	−49.30 d	145.53 E

Caption: DLS: dynamic light scattering. Values followed by equal letters in the same column did not differ statistically (*p* < 0.05) by Fisher’s test.

**Table 2:** Percentage values of the crystallinity index of AMKP and AMWP pulps and cellulose nanocrystals isolated by sulfuric acid hydrolysis at 50% (CNC-50), 52% (CNC-52), 54% (CNC-54), 56% (CNC-56), and 58% (CNC-58).

Sample	Crystallinity Index (%)
AMKP	33.22
AMWP	37.42
CNC-50	48.28
CNC-52	62.93
CNC-54	71.66
CNC-56	76.80
CNC-58	81.76

## Data Availability

Data are contained within the article.
